# Biochar is colonized by select arbuscular mycorrhizal fungi in agricultural soils

**DOI:** 10.1007/s00572-024-01149-5

**Published:** 2024-05-17

**Authors:** Patrick Neuberger, Carlos Romero, Keunbae Kim, Xiying Hao, Tim A. McAllister, Skyler Ngo, Chunli Li, Monika A. Gorzelak

**Affiliations:** 1 Lethbridge Research and Development Centre, Agriculture and Agri-Food Canada, 5403 - 1st Ave. S, Lethbridge, AB T1J 4B1 Canada; 2 Department of Chemistry and Biochemistry, University of Lethbridge, 4401 University Dr W, Lethbridge, AB T1K 3M4 Canada; 3 Division Forest, Nature and Landscape, KU Leuven Campus Geel, Geel, Belgium

**Keywords:** Soil microbiomes, Soil-derived AMF, Manure, Amplicon sequencing, Quinoa, Paraglomus

## Abstract

Arbuscular mycorrhizal fungi (AMF) colonize biochar in soils, yet the processes governing their colonization and growth in biochar are not well characterized. Biochar amendment improves soil health by increasing soil carbon, decreasing bulk density, and improving soil water retention, all of which can increase yield and alleviate environmental stress on crops. Biochar is often applied with nutrient addition, impacting mycorrhizal communities. To understand how mycorrhizas explore soils containing biochar, we buried packets of non-activated biochar in root exclusion mesh bags in contrasting agricultural soils. In this greenhouse experiment, with quinoa (*Chenopodium quinoa*) as the host plant, we tested impacts of mineral nutrient (as manure and fertilizer) and biochar addition on mycorrhizal colonization of biochar. *Paraglomus* appeared to dominate the biochar packets, and the community of AMF found in the biochar was a subset (12 of 18) of the virtual taxa detected in soil communities. We saw differences in AMF community composition between soils with different edaphic properties, and while nutrient addition shifted those communities, the shifts were inconsistent between soil types and did not significantly influence the observation that *Paraglomus* appeared to selectively colonize biochar. This observation may reflect differences in AMF traits, with *Paraglomus* previously identified only in soils (not in roots) pointing to predominately soil exploratory traits. Conversely, the absence of some AMF from the biochar implies either a reduced tendency to explore soils or an ability to avoid recalcitrant nutrient sources. Our results point to a selective colonization of biochar in agricultural soils.

**Supplementary Information:**

The online version contains supplementary material available at 10.1007/s00572-024-01149-5.

## Introduction

Arbuscular mycorrhizal fungi (AMF) colonize most terrestrial plants, including most domesticated crops (Smith and Read 2008). AMF form a nutritional symbiosis wherein the plant provides photosynthates and acts as the sole carbon source for the fungus, in exchange for soil-derived mineral nutrients furnished by the fungi (Parniske 2008). These fungi expand the volume of soil which plants may explore for nutrients, mainly phosphorus and nitrogen to a lesser extent, to trade to their host plant. Under low nutrient conditions, mycorrhizal plants may transfer an elevated proportion of carbon resources to symbiotic AMF to promote AMF phosphorus acquisition, a more resource efficient strategy than constructing additional roots or root hairs (Andrino et al. 2020). Sustainable agricultural management practices – such as no-till and reduced fertilization – can increase AMF abundance in soils by allowing for expanding mycelial networks and promoting root colonization (Fitter et al. 2011). In addition, integration of AMF into cropping systems also can improve: salt-tolerance (Saxena et al. 2017), drought tolerance (Tang et al. 2022), and disease resistance of host plants (Song et al. 2015), thereby improving resilience in the face of climate change. Sustainable cropping systems, promoting AMF, and growing plants tolerant to adverse conditions can help us produce food in increasingly adverse environments.

Quinoa (*Chenopodium quinoa* Willd.) is a seed-crop of increasing importance in North America as it is salt, drought, and frost tolerant (Ruiz et al. 2013). Increased interest in growing quinoa grain in Canada has fueled recent agronomic research into optimal growing conditions at Northern latitudes, although no work has been conducted on quinoa AMF in Canada (Nurse et al. 2016). While quinoa has previously been categorized as non-mycorrhizal, a number of recent studies have shown AMF colonization of quinoa roots, although there is reduction of AMF following quinoa rotations and colonization rates have been reported as diminished in subsequent crops (Urcelay et al. 2011; Wieme et al. 2020; Kellogg et al. 2021). Furthermore, AMF inoculation of quinoa plants has been shown to improve physiological markers (such as chlorophyll content and vegetative growth), improve response to stress, and improve soil health following harvest, in comparison to non-inoculated quinoa plants (Benaffari et al. 2023). Quinoa is deeply rooted, however, and in many studies symbiotic association of AMF with quinoa has been variable, with negligible colonization or colonization rates lower than for other economically important crops (Urcelay et al. 2011). Colonization with AMF in this plant family may follow a pathogenic root response including phytoalexin production which could nevertheless prime the plant, resulting in some of the benefits observed (Yactayo-Chang et al. 2020). Research into quinoa mycorrhizae is still in its infancy, and further research into AMF associating with quinoa may be beneficial to meeting the needs of this growing market.

Biochar benefits soil health through increasing nutrient availability as well as improving water retention and soil structure, through decreased bulk density (Lehmann et al. 2011; Palansooriya et al. 2019). Depending upon the physical characteristics of the biochar, it can have two important overall applications to agriculture: improving crop yield and increasing soil organic matter content. Many studies suggest positive effects of biochar on yield in meta-analyses, with application rates ranging from 5 to 20 Mg ha-1, although lower application rates can be used in combination with fertilization (< 1 Mg ha-1) (Joseph et al. 2021). Application of biochar has improved quinoa yields under both drought (Yang et al. 2020) and salinity-stressed conditions (Abbas et al. 2022), possibly by adsorbing excess Na^+^ and improving water retention. In addition, the agronomic importance of biochar can be supplemented through its ability to sequester carbon in arable soils, with an ability to reduce net greenhouse gas emissions by 1.8 Pg CO2-C annually without sacrificing food security (Woolf et al. 2010). Reduced net greenhouse gas emissions through biochar addition operates through increased methanotrophy, reduced N_2_O emissions as well as through the storage of recalcitrant carbon within biochar itself (Woolf et al. 2010). Repeated applications of biochar in arable soils builds soil organic carbon (SOC) stocks, whereas other unpyrolyzed carbon additions would be regularly decomposed, resulting in carbon mineralization (Joseph et al. 2021). Therefore biochar amendment is a promising avenue to improve crop yields while promoting sustainable agriculture and environmental benefits.

The capacity of AMF to colonize soil and biochar likely is influenced by nutrient availability and soil structure (George et al. 1995). Because of biochar’s ability to act as a phosphorus source (Glaser and Lehr 2019) as well as its heterogeneous structure, it is likely to represent a distinctive microhabitat for AMF. Indeed, previous research has shown enrichment of AMF in response to biochar addition, which authors posit may be attributable to the physical properties of biochar (Jin 2010). Arbuscular mycorrhizal fungi have been shown to colonize biochar, their hyphae penetrating small (< 10 μm) micropores and translocating nutrients (Hammer et al. 2014). Plants colonized by a single strain of arbuscular mycorrhizal fungus and supplemented with biochar have been reported to gain a productivity boost (Hammer et al. 2015). Soils, however, contain a mix of many different AMF with plants becoming colonized by a consortium of fungi, usually representative of the available inoculum in soils. Agricultural soils are also often amended with nutrients, both organic and inorganic as well as biochar. A number of mechanisms have been proposed to explain the increased relative abundance of AMF in soils amended with biochar, including changing the nutrient profiles of soil, altering AMF-microorganism interactions, altering AMF-plant interactions, and providing refugia for colonizing AMF (Warnock et al. 2007). Here, we conducted a greenhouse experiment wherein we buried packets of biochar in root-exclusion mesh bags to assess AMF hyphal colonization in arable soils from multiple locations, and we amended those soils with manure or fertilizer using quinoa as the host plant. To our knowledge, an examination of the colonization of biochar by naturally-occurring AMF from contrasting soils with different amendments, has not been done. We hypothesize that AMF diversity and community composition in pure biochar will differ from the surrounding soil because of differences in nutrient availability, chemical composition, and structure between the two.

## Methods

### Soils and amendment characterization

The top 15 cm layer of soils was collected in May 2018 from four long-term cropping field sites across Alberta, Canada: (i) a Dark Gray Luvisol from Beaverlodge (55°12'01"N, 119°23'51"W), (ii) an Orthic Brown Chernozem from Vauxhall (50°04'11"N, 112°0529"W), (iii) an Orthic Black Chernozem from Olds (51°43'46"N, 113°57'42"W), and (iv) an Orthic Brown Chernozem from Cranford (49°45'51"N, 112°20'31"W). All soils were deep and well-drained, and derived from glaciofluvial or glaciolacustrine deposits (Alberta 2016) and cropped to wheat (*Triticum aestivum* L.) in Beaverlodge, potato (*Solanum tuberosum* L.) in Vauxhall and Cranford, and barley (*Hordeum vulgare* L.) in Olds. Biochar was produced from pinewood (*Pinus* spp.) utilizing Engineered Biocarbon™ technology, i.e., a front-end biomass pyrolysis (< 650 °C) followed by a patented post-pyrolysis treatment step (Cool Planet Energy Systems, Inc., Greenwood Village, CO). The material was characterized by a surface area of 152 m^2^ g^− 1^, an ash content of 1.7%, a bulk density of 122 kg m^− 3^ (dry mass basis), and a volatile matter content of 25.4% (dry mass basis) (InnoTech Alberta Inc., Vegreville, AB). Manure was collected from cattle housed in a tie-stall barn. The material contained an average water content of 77–79% and resulted from a diet of 60% barley silage, 35% barley grain, and 5% standard supplement. Selected soil, biochar, and manure chemical properties are presented in Table 1.

**Table 1:** Selected soil, biochar, and cattle manure chemical properties. Means are presented with standard errors (*n* = 4)

	pH^a^	EC^a, b^ dS m^− 1^	Total N g kg^− 1^	Total C g kg^− 1^	C/N ratio	Total P^c^ g kg^− 1^
Soils						
Beaverlodge	5.25 ± 0.02	0.21 ± 0.01	2.63 ± 0.26	29.09 ± 2.86	11.16 ± 0.69	--^d^
Vauxhall	7.44 ± 0.11	0.75 ± 0.06	2.14 ± 0.03	22.13 ± 0.37	10.33 ± 0.02	--
Olds	7.39 ± 0.00	0.47 ± 0.02	4.65 ± 0.08	55.04 ± 0.81	11.84 ± 0.18	--
Cranford	7.83 ± 0.11	0.25 ± 0.01	2.23 ± 0.05	30.17 ± 2.01	13.19 ± 0.18	--
Amendments						
Biochar	7.17 ± 0.02	0.29 ± 0.01	1.58 ± 0.03	686.40 ± 2.50	444.74 ± 11.97	0.15 ± 0.01
Manure	7.23 ± 0.04	1.61 ± 0.03	20.97 ± 0.64	462.20 ± 3.40	22.11 ± 0.84	9.06 ± 0.27

^a^ Measured in a 2:1 water: sample slurry

^b^ EC, electrical conductivity

^c^ Determined by wet acid digestion with H_2_SO_4_ and H_2_O_2_ (Parkinson and Allen 1975)

^d^ Not determined

### Experimental design

A greenhouse experiment was conducted at the Lethbridge Research and Development Centre of Agriculture and Agri-Food Canada (Lethbridge, AB). Each nursery pot (4-L) was filled with 3 kg of air-dried, sieved soil (< 2 mm). Amendments were manually applied at a rate of 3.0 Mg ha^− 1^ (biochar), 200.0 Mg ha^− 1^ and 3.0 Mg ha^− 1^ (manure + biochar, respectively), and 150 kg N ha^− 1^ [(NH_4_)_2_SO_4_], 50 kg P ha^− 1^ (KH_2_PO_4_) and 3.0 Mg ha^− 1^ (fertilizer-NP + biochar, respectively) generating four experimental treatments for each soil type, i.e., un-amended control (C), biochar (B), biochar + manure (B + M), biochar + NP-fertilizer (B + F). Six nylon-sealed (35 μm mesh permitting AMF hyphal penetration (Friese and Allen 1991; Hempel et al. 2007; Błaszkowski et al. 2017) biochar packets (1.5 g, 3 × 3 cm) were buried at a depth of 5 cm inside each pot (except for C). Four replicate pots were prepared for each soil type x treatment combination and randomly arranged in the greenhouse. Eight seeds of quinoa cv. NQ94PT were sown in each pot on July 11, 2018. Plant density was reduced to four per pot two weeks after emergence. All pots were irrigated with distilled water during the experiment. The greenhouse was kept at 19 °C ± 0.5 for the duration of the experiment, with no added light. Quinoa was harvested on November 26, 2018, biomass was harvested, seed weight was recorded, and fresh soil from each pot was homogenized and sub-sampled for chemical analysis (50 g) or DNA extraction (5 g). Biochar was retrieved from packets and homogenized. DNA was extracted from soil and biochar stored at -20 °C and analyzed within a month of sample collection.

### Soil chemical analysis

Soil pH and EC were determined using a 2:1 (water: soil) slurry. Olsen P was determined by extracting 2.5 g of air-dried soil with 25 mL of 0.5 M NaHCO_3_ (Olsen et al. 1954). Concentrations were quantified by colorimetry with a discrete analyzer (EasyChem Pro, Systea Analytical Technology, Anagni, Italy). Water-extractable organic C [(WEOC); mg C kg^− 1^] and water-extractable total N [(WETN); mg N kg^− 1^] were quantified in syringe-filtered 15 mL aliquots (< 0.45 μm) using a TC and TN combustion analyzer (TOC-V_CSH_ and TNM-1 Shimadzu Corp., Kyoto, Japan) following the procedure of (Chantigny et al., 1999). A sub-sample of air-dried soil (< 2 mm) was ball-milled (< 0.15 mm) and used to determine total C (TC), total nitrogen (TN), ^15^N/^14^N isotope ratios (δ^15^N‰), and ^13^C/^12^C isotope ratios (δ^13^C‰) by dry combustion using a CN analyzer (NC2100, Carlo Erba Instruments, Milan, Italy) coupled with an Optima mass spectrometer (Micromass, Manchester, UK). NH_4_^+^-N and NO_3_-N were determined by extracting 5 g of soil with 25 mL of 2 M KCl and quantified by the modified indophenol blue technique (Sims et al., 1995) using a microplate spectrophotometer at 650 nm (Multiskan GO, Thermo Fisher Scientific, Waltham, MA).

### DNA extraction and sequencing

DNA was extracted from soil and biochar by using the Qiagen Powerlyzer Powersoil DNA extraction kit as per manufacturer protocols, combined with bead beating using a MP Biomedical Fast Prep Bead Beater (MP Biomedicals, Ohio, USA). DNA purity was confirmed using a Biodrop spectrophotometer and DNA concentrations determined using a Qubit v4 fluorometer (ThermoFisher Scientific, Massachusetts, USA). All samples were checked to ensure amplification using the AMF SSU primer pair NS31 (5’-TTGGAGGGCAAGTCTGGTGCC-3’) and AML2 (5’-GAACCCAAACACTTTGGTTTCC-3’) with the conditions outlined in Morgan and Egerton-Warburton (2017). Libraries were prepared and sequencing was performed by Genome Quebec using an Illumina MiSeq with V3 chemistry at 2 × 300 bp paired-end (PE) configuration (Illumina, San Diego, California, USA). Each PCR was conducted in a 7 µL reaction: 1X PCR Buffer with 18mM MgCl_2_ (Roche), 5% DMSO (Roche), 0.2 mM dNTP mix (NEB), 0.02 U/µL FastStart High Fi (Roche), 0.5 µM NS31 primer, 0.5 µM AML2 primer, 1 µL of 10-fold diluted DNA template and molecular grade water. Thermocycler conditions were as follows: denaturation at 94 °C for 3 min; 35 cycles of 94 °C for 45 s, 63 °C for 60 s, 72 °C for 90 s; and a final extension of 72 °C for 10 min. Sequence processing was performed in QIIME2 with Dada2 (Bolyen et al. 2019). 5’ ends of the forward reads were trimmed at 21 bp, and the 3’ ends of the reverse reads were trimmed at 22 bp, corresponding to a median QC over 20. Forward reads were then truncated to a max of 295 bp and reverse reads were truncated to a max of 283 bp. Adaptor sequences were removed using filterANDtrim. Sequences were then dereplicated, chimeras were removed, and amplicon sequence variants (ASVs) were resolved with Dada2 in QIIME2. Taxonomy was assigned using BLAST + with a trained MAARJAM v2 database to derive virtual taxon assignments (VTX) (Opik et al. 2010). Due to the high number of unknown assignments using VTXs, α-diversity and β-diversity measures were calculated using the original ASVs. Sequences were not rarefied. Prior to quality filtering, read counts ranged from 13,776 to 263,566 reads per sample for a total of 15,253,943 reads. After merging reads, 3,659,625 reads passed quality filtering (23% of the total read count). The average read count in the soil samples was 3,121 reads (with a maximum of 21,582 reads) and the average read count in the biochar samples was 1,367 (with a maximum of 22,085 reads). A total of 2886 AMF ASVs were identified. All sequences identified matched to Glomeromycota, with 88.7% of these supporting classification past the order level.

### Statistical analysis

Statistical analysis was performed in R v3.6.4 (R Development Core Team, 2008). Diversity analyses were conducted in the Phyloseq R package and included α-diversity (richness, Pielou’s evenness, and Shannon diversity) and β-diversity metrics, including Bray-Curtis distance matrices (McMurdie and Holmes 2013). Normalization of ASV counts for β-diversity was undertaken using a variance stabilizing transformation implemented in the DeSeq2 R package (Love et al. 2014). The β-diversity metrics were visualized in non-metric multidimensional scaling (NMDS) and dbRDA plots in vegan (Oksanen et al. 2019). Soil chemical properties were used as explanatory variables to determine their effects on AMF community composition. Forward selection of environmental variables was used to reduce multicollinearity of the model. The vegan R package was used to determine the significance of β-diversity differences with PERMANOVAs and correlations with environmental variables using the mantel test (Oksanen et al. 2019). Count data were not normalized for α-diversity or relative abundance analysis. Linear mixed models were constructed (LMM; Method = REML) for comparisons of α-diversity between soil sites (Beaverlodge, Vauxhall, Olds, and Cranford; within either bulk soil or biochar packets), between nutrient amendments (F, B + M, and B + F; within either bulk soil or biochar packets), and between Bulk Soil vs. Biochar packets themselves. The homogeneity of variance, normality and outliers of the residuals were assessed in DHARMa. Data were square-root transformed where appropriate to correct for non-normality and heteroscedasticity in the R package MASS. Significant differences in α-diversity were assessed using a two-way ANOVA with a Tukey post-hoc test when appropriate in R. Relative abundance tables were made using the Phyloseq package in R and stacked bar graphs were created using ggplot2 (Wickham 2016). All β-diversity, α-diversity, relative abundances, and correlation analyses were performed using ASV data. Spearman correlations between α-diversity metrics and environmental parameters were conducted in corrplot v0.92. Multiple comparison corrections were performed using a Benjamini-Hochberg multiple comparisons method.

## Results

PERMANOVA analysis showed that community composition differed in site (*P* < 0.001), sample type (soil or biochar packet) (*P* < 0.001), and amendment (*P* < 0.001), with substantial differences in taxonomy, particularly across sample types (Supp. Table 1). Average richness across all samples was 39.74 ± 22.45, average Pielou’s evenness was 0.49 ± 0.16, and average Shannon diversity was 1.76 ± 0.62. Across all soils, richness negatively correlated with net seed dry weight (*r* = -0.37, *P* = 0.033) and δ^13^C‰ (*r* = -0.41, *P* = 0.033), and Shannon diversity was negatively correlated with net seed dry weight (*r* = -0.31, *P* = 0.001). Community composition was significantly correlated with EC, TC, TN, WETN, and total quinoa biomass according to Mantel tests, although all correlations were weak (Supp. Table 2). Most sequences belonged to Paraglomerales (78%), with 18% belonging to Glomerales, 4% belonging to Archeosporales, and < 1% belonging to Diversisporales. Across soil and biochar samples, 18 VTs were observed, three of which, VTX0039, VTX00155, and VTX00419, were unique to the biochar samples (Supp. Table 3). The most abundant VTs were VTX00348 (82.7%), VTX00444 (10.2%), VTX00067 (2.4%), and VTX00004 (1.1%) the first two of which belonged to Paraglomerales and were found in both soil and biochar. VTX00348 was the single most abundant VT in all sites, in both soil and biochar samples, and in all soil amendments except for B + F. Most VTs were below 5% relative abundance.

### AMF communities are distinct between each soil type

**Fig. 1:**
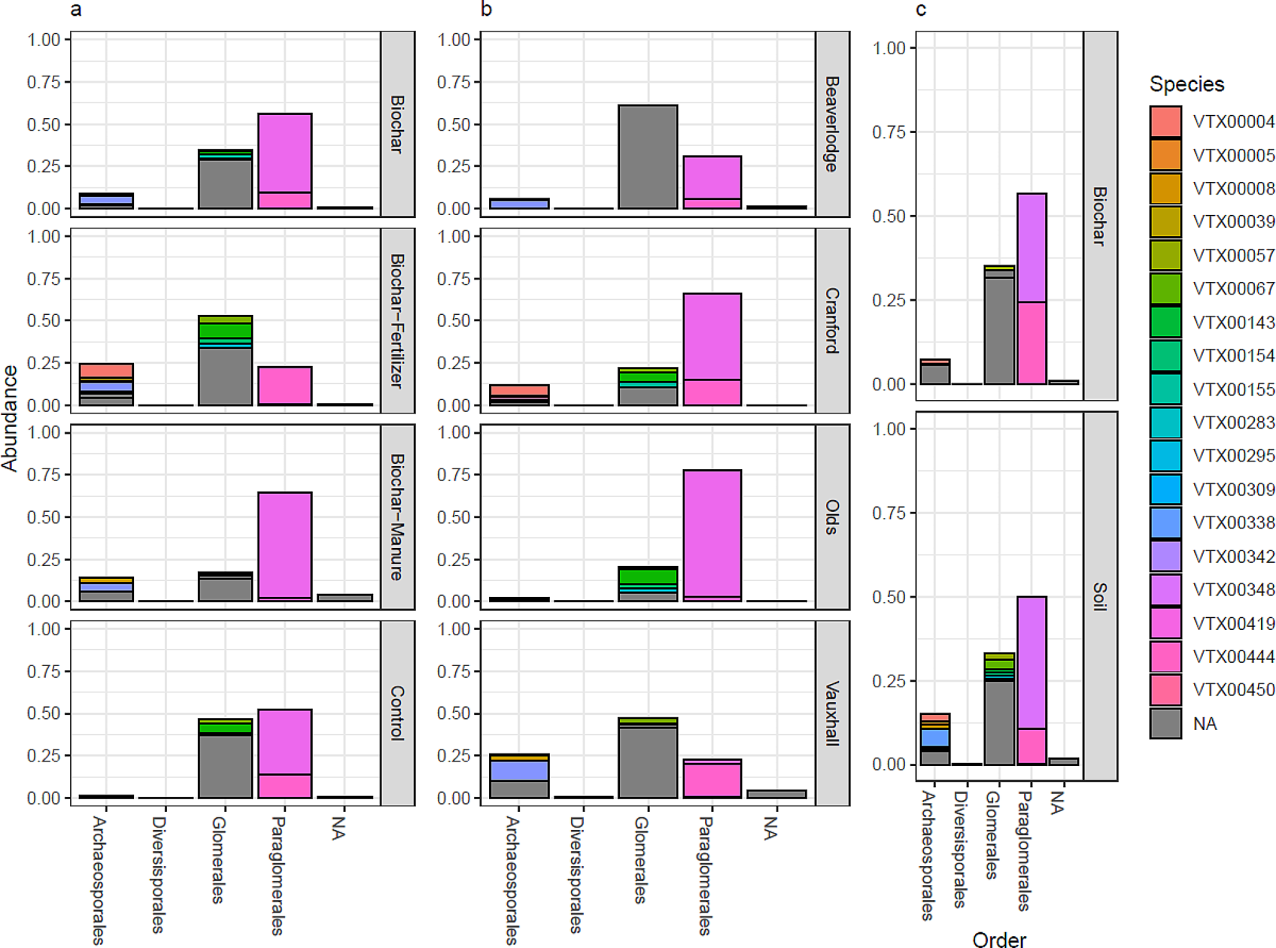
Relative abundances of AMF virtual taxa from bulk greenhouse soils and biochar packets combined across class level between (**a**) treatment, (**b**) soil type, and (**c**) sample type. Treatment groups include soils treated with biochar, fertilizer, biochar with fertilizer, and an untreated control. Soil types describe the geographic source of the soil used in the greenhouse study, including Beaverlodge, Cranford, Olds, and Vauxhall, all of which are found in Alberta, Canada. The sample type refers to the soil and biochar compartments within each pot. NA indicates unassigned species level ASVs. Only main effects are illustrated

Diversity, and community composition differed significantly across sites. Main effects in relative abundance show differences between soil types, treatments, and illustrate the differences of AMF identified in biochar packets (Fig. 1). Glomerales had higher relative abundance in Beaverlodge and Vauxhall than in Cranford and Olds soils (Fig. 1).

**Fig. 2:**
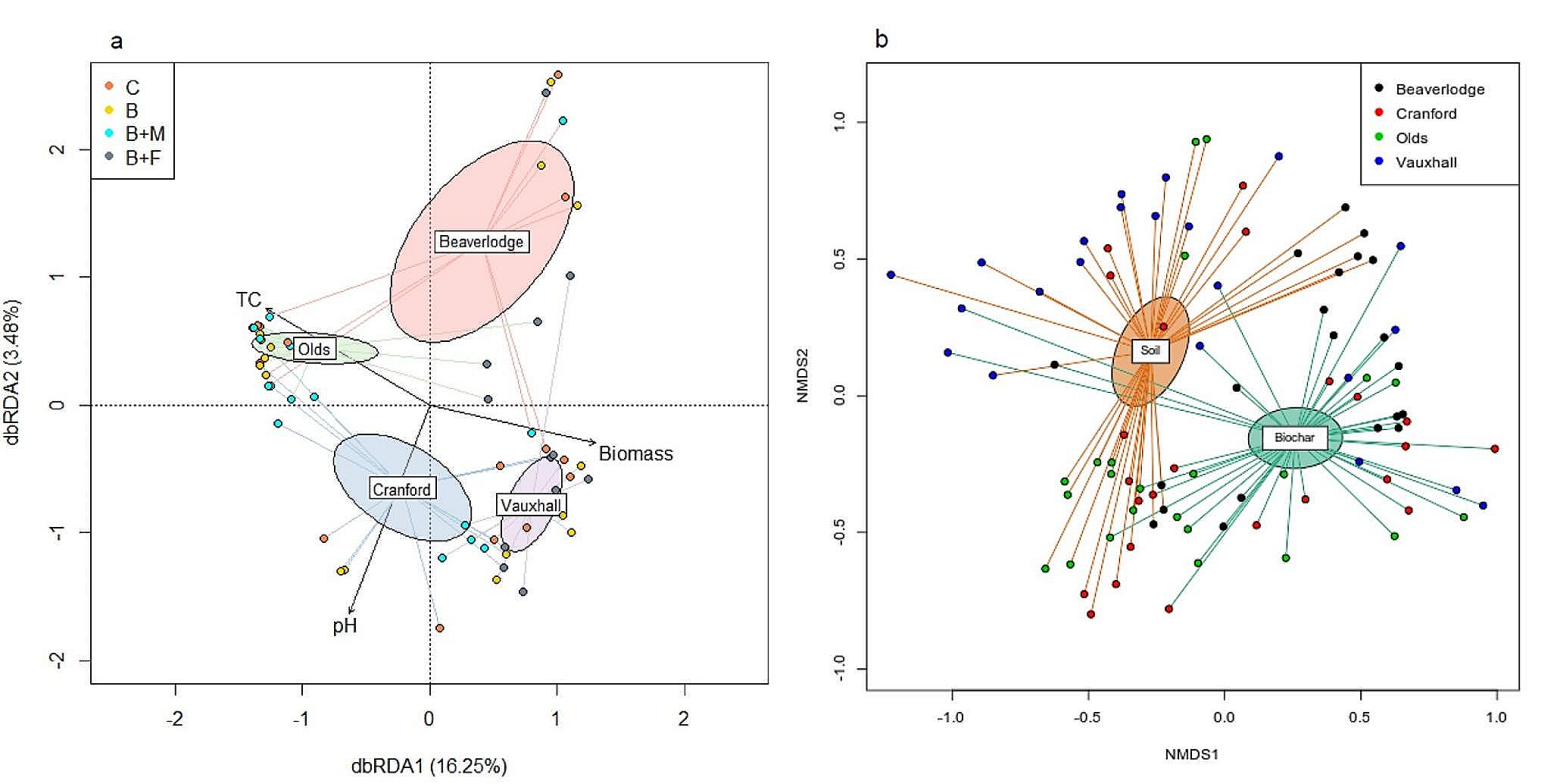
Ordinations of (**a**) soil samples and (**b**) soil samples compared to biochar samples. (**a**) Distance based redundancy analysis (dbRDA) of significant environmental parameters and microbial communities. Standard error ellipses represent Beaverlodge (red), Cranford (blue), Olds (green), and Vauxhall (purple). Environmental parameters which significantly improved the model are visualized as vectors with black arrows: TC (Total Carbon), pH, Biomass (Total Plant Biomass). Soil amendments are visualized as C (Control; coral), B (Biochar; yellow), B + M (Biochar and Manure; cyan), B + F (Biochar and Fertilizer; grey). An ANOVA showed that the model was significant (F = 3.9967, *P* = 0.001) and both axes dbRDA 1 (F = 11.2125, *P* = 0.001) and dbRDA 2 (F = 2.4002, *P* = 0.006) were significant. Ellipses represent the standard deviation of variation within each group, centered at the group centroid. (**b**) Non-metric multidimensional scaling plot (NMDS) of AMF community composition between biochar packet and bulk soil sample types. Community composition changes significantly between biochar and soils (PERMANOVA, *p* < 0.05). Ellipses represent the standard deviation of variation within each group, centered at the group centroid

Paraglomerales were most abundant in all soils and biochar packets, and were reduced with the application of NP-fertilizer amended biochar to soils. Every soil type harbored a distinct AMF community (*P* = 0.001) (Supp. Table 4). Community differences were supported by dbRDA ordination plots (Fig. 2a). Vauxhall soils had higher Pielou’s evenness than Olds soils (*P* = 0.01) and Beaverlodge (*P* = 0.04), and Vauxhall soils had higher Shannon diversity than Olds (*P* = 0.01) and Beaverlodge (*P* = 0.01). All soil and yield parameters correlated with AMF community composition (TN, TC, WETN, EC, and biomass etc.) with the exception of NH_4_^+^ and whole plant tissue weight (including seeds; Supp. Table 2). The environmental parameters with the most explanatory power regarding AMF community composition were TC, Biomass, and pH (Fig. 2a). Indicator species analysis identified 0, 4, 4, and 4 unique indicator ASVs within Beaverlodge, Cranford, Olds, and Vauxhall soils, respectively (*P* < 0.05) (Supp. Table 5).

**Table 2:** Alpha-diversity of compartments, both soil and biochar. All metrics were calculated using ASV counts. Means are represented with their standard error. Means followed by the same letter do not differ significantly by ANOVA (*P* < 0.05)

		Richness	Shannon	Evenness
Sample Type	Biochar	29.87 ± 1.39 (a)	1.59 ± 0.09 (a)	0.47 ± 0.02 (a)
	Bulk Soil	50.20 ± 3.9 (b)	1.91 ± 0.1 (b)	0.5 ± 0.02 (a)

**Table 3:** Alpha diversity metrics using ASVs of soil samples with their corresponding Site and Treatment. Treatments are listed as B (Biochar), B + F (Biochar and Fertilizer), B + M (Biochar and Manure), and C soils. Means are presented with their standard error. Means within a column followed by the same letter do not differ significantly by an ANOVA (*P* < 0.05) within their respective comparison (Soils: Site vs. Treatment; Biochar: Site vs. Treatment)

			Richness	Shannon	Pielou’s Evenness
Soils	Site	Olds	43.87 ± 4.5 (a)	1.59 ± 0.13 (a)	0.43 ± 0.03 (a)
		Cranford	48.88 ± 8.17 (a)	2.01 ± 0.15 (ab)	0.54 ± 0.04 (ab)
		Beaverlodge	38.75 ± 6.03 (a)	1.61 ± 0.18 (a)	0.45 ± 0.04 (a)
		Vauxhall	56.93 ± 7.62 (a)	2.3 ± 0.11 (b)	0.59 ± 0.03 (b)
	Treatment	B	43.88 ± 6.83 (a)	1.84 ± 0.17 (a)	0.5 ± 0.04 (a)
		B + F	54.92 ± 7.52 (a)	2.48 ± 0.11 (b)	0.64 ± 0.02 (b)
		B + M	53 ± 5.93 (a)	1.54 ± 0.12 (a)	0.39 ± 0.03 (a)
		C	39.29 ± 7.11 (a)	1.85 ± 0.14 (a)	0.52 ± 0.04 (a)
Biochar	Site	Beaverlodge	32 ± 3.98 (a)	1.57 ± 0.13 (a)	0.46 ± 0.03 (a)
		Cranford	42.13 ± 4.13 (a)	1.84 ± 0.15 (ab)	0.5 ± 0.04 (ab)
		Olds	38.83 ± 3.75 (a)	1.5 ± 0.12 (a)	0.42 ± 0.03 (a)
		Vauxhall	45.77 ± 6.56 (a)	2.07 ± 0.11 (b)	0.57 ± 0.02 (b)
	Treatment	B	36.45 ± 3.77 (a)	1.57 ± 0.11 (a)	0.45 ± 0.02 (a)
		BF	39.89 ± 4.66 (a)	2.03 ± 0.13 (b)	0.57 ± 0.03 (b)
		BM	43.1 ± 3.9 (a)	1.66 ± 0.1 (ab)	0.46 ± 0.03 (a)

### Biochar colonization

While ASVs were used for β-diversity and α-diversity metrics, sequences were also taxonomically assigned. Within the ASV dataset, only 12 VTXs were identified in biochar packets, including two VTXs which were not observed in the soils (Glomus VTX00419 and Glomus VTX00155) compared to 17 in soil samples (Fig. 1c). Consequently, AMF communities differed between soil and biochar packets, with significantly lower richness and diversity in biochar packets (Fig. 2b; Table 2; Suppl. Table 6). To ensure PERMANOVA results were attributable to community composition differences and not dispersion differences, a betadispersion analysis was performed, showing that dispersion did not differ significantly between soil and biochar compartments (*P* = 0.5345). An average of 29.87 ASVs were present in biochar, composed primarily of Paraglomerales taxa, while an average of 50.20 ASVs found in the surrounding soils (Table 2) primarily comprised Glomerales.

Differential abundance analysis showed 14 Glomerales ASVs which were most abundant in bulk soil samples, while 6 Paraglomerales ASVs were most abundant in biochar packets.

### Amendment treatments

While amendment likely contributed to community compositional differences, there were no significant differences in community composition except between B + M and B + F (*P* < 0.001). Community composition of all soil amendments resembled those of the C soils (*P* > 0.05). Ordination revealed no reliable trend between soil amendment and community composition (Fig. 2a; Suppl. Table 7). Amendments had no impact on observed AMF ASV richness, however fertilizer addition increased Shannon diversity and Pielou’s evenness (Table 3). All nutrient additions increased Archaeosporales relative abundance versus the C treatment (Fig. 1). B + F increased Glomerales relative abundance and decreased Paraglomerales abundance relative to the C treatment (Fig. 1).

## Discussion

Naturally occurring *Paraglomus* appear to predominately colonize non-activated biochar (Fig. 1). Despite differences in AMF communities among soil types acquired from distant locations with distinct characteristics (Table 1), we nonetheless found select AMF in biochar packets across these soil types and across treatments (Fig. 1). We found two *Glomus* and one *Gigaspora* virtual taxa exclusively in the biochar packets (Suppl. Table 3) and never in the soil samples. Because AMF are obligate biotrophs and require a host plant to proliferate with the fungus growing towards the biochar through the soil while actively deriving all carbon directly from quinoa root cells, it is unlikely that these were absent from the soils. Rather, in all likelihood, we did not sample intensively enough to capture these three virtual taxa in soil. Of the 19 virtual taxa found in soils, 12 also were found in biochar showing that 7 virtual taxa were not represented in the biochar. This observation could represent AMF with traits which do not form long hyphal strands throughout soil and thus did not extend far enough to colonize the packets. The AMF absent from biochar also might have the capacity to detect nutrients, and detecting none (the biochar was non-activated, meaning no nutrients were added) (Table 1), they explored other patches of soil that did contain nutrients. Finally, our sampling effort might have missed detecting these AMF in biochar. Nevertheless, the edaphically distinct soils used in this study harboured distinct AMF communities (Fig. 2) and those communities shifted with amendment addition.

Despite differences in AMF community composition between soil types and amendments, we found *Paraglomus* preferentially colonized biochar. The relative abundance of Paraglomeraceae may be unaffected by or increased in non-activated biochar packets, suggesting it may display unique hyphal exploratory traits. However, it is critical to note that high throughput sequencing data is compositional, and therefore an increase in Paraglomeraceae abundance may instead reflect the reduced abundance of other AMF taxa. AMF life history traits include colonizing ability, dispersal ability, stress tolerance, disturbance tolerance, reproduction versus vegetative growth investment, and reproductive mode (Hart and Reader 2002; Powell et al. 2009; Horsch et al. 2023). Paraglomus may have a life history strategy that includes investment into hyphal exploratory structures (absorptive hyphae, runner hyphae and hyphal bridges) over the formation of more internal root colonization structures (infection units, hyphae, contact points) (Hart and Reader 2004). Paraglomeraceae are reported to be largely absent from plant roots and AMF spore communities, while also forming the most abundant taxonomic group within soils (Hempel et al. 2007). Therefore, it is possible that the differences seen here between soil and biochar communities may be attributable to *Paraglomus* life history strategies. While many taxonomic groups invest more energy into external structures, (including Archaeosporales, Diversisporaceae, and Acaulosproraceae) only Paraglomeraceae was enriched in our biochar packets. Furthermore, the biochar used in this study was not activated, that is, no nutrients were added to the packets prior to the experimental setup. The non-activated biochar had a higher C: N ratio than the surrounding soil, representing an environment with lower nitrogen content (Table 1). Thus, Paraglomeraceae is either stimulated by low nitrogen conditions or more likely Paraglomeraceae constitutively explore soil without the capacity to detect and alter hyphal exploration in response to nutrient patches.

Biochar represents a unique habitat of rough porous materials with large amounts of aromatic carbon which is capable of harbouring AMF within small microsites (Warnock et al. 2007; Romero et al. 2021). Incubation experiments of biochar with soil microbial communities have identified microbial oxidation of biochar, both in the presence and absence of soil (Kuzyakov et al. 2009; Zimmerman 2010). Phosphate-solubilizing bacteria (PSB) attached to AMF hyphae facilitate metabolism and uptake of phosphate by AMF (Sharma et al. 2020). Therefore, bacterially mediated degradation of biochar may be facilitated by AMF colonization of biochar. This degradation may take the form of carbon oxidation, and/or phosphate-solubilization from biochar, liberating phosphorus and recalcitrant carbon compounds from biochar to AMF and the surrounding soil. Thus, synergism between PSB and AMF might contribute to the degradation of biochar and increased availability of nutrients to plant communities.

Alternatively, the host plant quinoa may preferentially associate with Paraglomeraceae and therefore enrich it over other AMF species present in the soils. Quinoa associates with AMF (Wieme et al. 2020), however, to our knowledge, a preference for Paraglomeraceae has not been reported. AMF colonization of quinoa roots was found to be lower than that of other crops such as wheat, chickpea (*Cicer arietinum*), and barley (Wieme et al. 2020). Furthermore, Cai et al. (2020) found bacterial and fungal community diversity associated with quinoa increased with elevation. They suggested that root associating fungal communities were deterministic with respect to edaphic characteristics. González-Teuber et al. (2017) reported that quinoa was significantly affected by the presence of endophyte fungi, and surmised that quinoa may benefit in drought conditions from endophytic associations. Conversely, (Urcelay et al. 2011) found no AMF colonization in quinoa with the presence of a pathogenic root fungus. Future studies should determine the extent of root colonization of quinoa by local AMF within Canadian soils.

*Paraglomus* has been found to be selected by other plants. For example, (Xiao et al. 2014) found that the growth of the invasive plant *Chromolaena ordorata* resulted in increased *Paraglomus* in soils, correlating with improved competitive outcomes for the plant. Plants exposed to high-stress environments may depend on *Paraglomus* for water retention and nutrients (Zhang et al. 2019). *Paraglomus* relative abundance increases in cropping systems with low soil pH (Dai et al. 2014), is widespread throughout agricultural soils (Gosling et al. 2014), and occupies low pH niches globally (Davison et al. 2021). However, Gosling et al. (2014) found *Paraglomus* associated more often with organic than with conventional farming practices.

The relationships between AMF community composition, diversity, and abundance with P and N availability have been well documented (George et al. 1995; Treseder and Allen 2002; Johnson 2010; Qiu et al. 2022). Biogeographic studies have identified strong influences on AMF diversity of high temperature, low C, low N pressing community composition in one direction and high precipitation, low pH, low K, and low P pressing community composition in the other direction (Davison et al. 2021). This study supports such findings, showing that distinct communities from soils sourced across a wide geographic range were associated with a strong pH gradient and differed considerably in nutrient content (Table 1). Thus soil physico-chemical parameters appeared to influence AMF community composition in our study. Similar to other studies, N content in soils can shift AMF communities and can decrease AMF diversity (Zhang et al. 2021). As AMF abundance and diversity increase in low nutrient conditions, increased sporulation and spore-carrying hyphal structures are likely an indication of the stimulation of AMF by host plants in response to lack of adequate nutrients. This is supported within our dataset wherein soils with low N and P availability also exhibited elevated richness and a distinct community structure from other soils; these also were associated with diminished Glomeralesrelative abundance. It could be that what we missed observing Glomerales that was in roots, as it is often more abundant in roots than in soil (Hart and Reader 2002). Interestingly, NP-fertilizer addition decreased Paraglomerales, but increased the Glomerales relative abundances, whereas Paraglomerales abundance increased with manure addition, while *Glomus* abundance decreased with manure addition, suggesting an opposing relationship with a complex carbon-rich nutrient amendment. Sheldrake et al. (2018) showed that removing litter as a complex nutrient source shifted AMF community composition in soils, and (Elzobair et al. 2016) showed significant changes in AMF abundance with biochar and manure amendment. Our findings build upon these previous works, suggesting that AMF community composition can also be affected by nutrient amendments known to alter AMF absolute abundance.

## Conclusion

We suggest that quinoa is able to associate with a variety of AMF found across arable soils in western Canada. Paraglomeraceae predominately colonizes non-activated biochar and this may represent hyphal exploratory traits unique to the taxon. Biochar was colonized by select naturally occurring soil-derived AMF with site of soil collection a strong indicator of AMF community composition.

### Electronic supplementary material

Below is the link to the electronic supplementary material.

Supplementary Material 1

## Data Availability

Raw sequences are available on the EMBL Nucleotide Sequence Database through accession PRJEB75442.
