# Supplementary material for: Biochar is colonized by select arbuscular mycorrhizal fungi in agricultural soils
Source: Mycorrhiza. 2024 May 17;34(3):191–201. doi: 10.1007/s00572-024-01149-5 (PMC11166811; doi:10.1007/s00572-024-01149-5)
Supplement: Supplementary file 1 — Supplementary Material 1 [file 572_2024_1149_MOESM1_ESM.docx]

# Supplementary Figs and Tables

Supplementary Table 1. PERMANOVA of Bray-Curtis dissimilarity matrix constructed from AMF communities as visualized in Fig 2, showing significant impact of treatment, soil type, and a significant interaction effect between the two treatments. Soil Type: soils originating from Cranford, Vauxhall, Beaverlodge, or Olds. Treatment: Control; B: Biochar; B+M: Biochar and Manure; B+F: Biochar and Fertilizer. Df: degrees of freedom; SumsOfSqs: Sum of squares; F.Model: F value by permutation; R2: coefficient of determination; Pr(>F): p-values based on 1000 permutations (the lowest p-value being 0.001).

|  | Df | SumsOfSqs | MeanSqs | F.Model | R2 | Pr(>F) |
| --- | --- | --- | --- | --- | --- | --- |
| Soil | 3 | 0.45525 | 0.151749 | 16.6769 | 0.40419 | 0.001 |
| Treatment | 3 | 0.12852 | 0.04284 | 4.7081 | 0.11411 | 0.001 |
| Soil:Treatment | 9 | 0.16037 | 0.017819 | 1.9583 | 0.14239 | 0.001 |
| Residuals | 42 | 0.38217 | 0.009099 |  | 0.33931 |  |
| Total | 57 | 1.12631 |  |  | 1 |  |

Supplementary Table 2. Summary of Mantel test results of environmental parameters, both edaphic and plant, and beta-diversity across soil samples (not biochar*soil sample groups). Euclidean distances of each environmental parameter were tested against Bray Curtis dissimilarity matrices of AMF community composition.

|  | Mantel r Stat | p value |
| --- | --- | --- |
| Net Seed Weight | 0.2899 | 1.00E-04 |
| Seed with Shell | 0.2794 | 1.00E-04 |
| ECdS | 0.2756 | 1.00E-04 |
| WET N | 0.2197 | 1.00E-04 |
| WEON | 0.212 | 1.00E-04 |
| NO3NH4N | 0.2088 | 1.00E-04 |
| CN Ratio | 0.1983 | 1.00E-04 |
| Biomass | 0.1805 | 2.00E-04 |
| delta 13C | 0.1471 | 5.00E-04 |
| delta 15N | 0.1161 | 0.0024 |
| TN | 0.1152 | 0.0023 |
| Olsen P | 0.09759 | 0.007 |
| TC | 0.09425 | 0.0066 |
| pH | 0.07835 | 0.0204 |
| WEOC | 0.06608 | 0.0391 |
| NH4N | 0.05341 | 0.0777 |
| Plant Tissue Weight | 0.03539 | 0.1213 |

Supplementary Table 3. Taxonomy and habitat of AMF VTXs. Habitat was chosen based upon the presence/absence of each VTX within the compartment type (Soil and/or Biochar).

| Order | Genus | Species | Habitat |
| --- | --- | --- | --- |
| Archaeosporales | Archaeospora | VTX00004 | Soil And Biochar |
| Archaeosporales | Archaeospora | VTX00005 | Soil |
| Archaeosporales | Archaeospora | VTX00008 | Soil And Biochar |
| Diversisporales | Gigaspora | VTX00039 | Biochar |
| Glomerales | Claroideoglomus | VTX00057 | Soil And Biochar |
| Glomerales | Glomus | VTX00067 | Soil |
| Glomerales | Glomus | VTX00143 | Soil |
| Glomerales | Glomus | VTX00154 | Soil And Biochar |
| Glomerales | Glomus | VTX00155 | Biochar |
| Glomerales | Claroideoglomus | VTX00193 | Soil |
| Archaeosporales | Ambispora | VTX00283 | Soil |
| Glomerales | Glomus | VTX00295 | Soil |
| Glomerales | Glomus | VTX00309 | Soil |
| Archaeosporales | Archaeospora | VTX00338 | Soil And Biochar |
| Glomerales | Glomus | VTX00342 | Soil And Biochar |
| Paraglomerales | Paraglomus | VTX00348 | Soil And Biochar |
| Glomerales | Glomus | VTX00419 | Biochar |
| Paraglomerales | Paraglomus | VTX00444 | Soil And Biochar |
| Archaeosporales | Archaeospora | VTX00450 | Soil And Biochar |

Supplementary Table 4. Pairwise PERMANOVA of a Bray Curtis dissimilarity matrix constructed from AMF communities between all soil types as visualized on Fig 2. F.Model: F value by permutation; R2: coefficient of determination; p.value: p-values based on 1,000,000 permutations (the lowest p-value being 1.00E-06); p.adjusted: p-values as adjusted using a Bonferroni correction method.

| Comparisons | F.Model | R2 | p.value | p.adjusted |
| --- | --- | --- | --- | --- |
| Olds: Cranf | 10.447961 | 0.2648543 | 7.00E-06 | 4.20E-05 |
| Olds: BV_acid | 11.988305 | 0.3241107 | 2.50E-05 | 1.50E-04 |
| Olds: VauxH | 16.484612 | 0.3705689 | 1.00E-06 | 6.00E-06 |
| Cranf: BV_acid | 12.324317 | 0.3215796 | 1.00E-06 | 6.00E-06 |
| Cranf: VauxH | 8.597526 | 0.2286727 | 1.00E-06 | 6.00E-06 |
| BV_acid: VauxH | 13.97428 | 0.3585513 | 1.00E-06 | 6.00E-06 |

Supplementary Table 5. Indicator species analysis of AMF ASVs which have been identified to the VTX level. Habitat: the soil type/amendment/sample type which each VTX is associated with; Indval: indicator value (IndVal = 0 shows no indication while Indval = 100 shows complete indication); p value: the significance of the IndVal.

| Indicator Species | Habitat | IndVal | p value |
| --- | --- | --- | --- |
| Soil Type |  |  |  |
| Glomus VTX00154 | Cranford | 0.642 | 0.003 |
| Archaeospora VTX00004 | Cranford | 0.612 | 0.002 |
| Archaeospora VTX00450 | Cranford | 0.592 | 0.006 |
| Ambispora NA | Cranford | 0.5 | 0.01 |
| Glomus VTX00067 | Olds | 0.734 | 0.001 |
| Glomus VTX00143 | Olds | 0.516 | 0.01 |
| Claroideoglomus VTX00193 | Olds | 0.447 | 0.035 |
| Glomus VTX00295 | Olds | 0.447 | 0.042 |
| Glomus VTX00309 | Vauxhall | 0.726 | 0.001 |
| Archaeospora VTX00338 | Vauxhall | 0.645 | 0.003 |
| Ambispora VTX00283 | Vauxhall | 0.516 | 0.012 |
| Gigaspora VTX00039 | Vauxhall | 0.447 | 0.032 |
| Glomeromycetes NA | Beaverlodge+Vauxhall | 0.657 | 0.022 |
| Paraglomus VTX00348 | Beaverlodge+Cranford+Olds | 0.792 | 0.007 |
| Paraglomus NA | Beaverlodge+Cranford+Vauxhall | 0.548 | 0.303 |
| Archaeospora VTX00008 | Beaverlodge+Olds+Vauxhall | 0.616 | 0.037 |
| Archaeosporales NA | Cranford+Olds+Vauxhall | 0.659 | 0.023 |
| Claroideoglomus VTX00057 | Cranford+Olds+Vauxhall | 0.626 | 0.045 |
| Soil Amendment |  |  |  |
| Archaeospora VTX00005 | Biochar+Fertilizer | 0.728 | 0.001 |
| Archaeospora VTX00008 | Biochar+Manure | 0.858 | 0.001 |
| Glomus VTX00309 | Biochar+Manure | 0.556 | 0.038 |
| Paraglomus VTX00348 | Biochar+Manure+Control | 0.834 | 0.001 |
| Sample Type |  |  |  |
| Archaeospora NA | Biochar | 0.412 | 0.047 |
| Archaeospora VTX00008 | Soil | 0.591 | 0.001 |
| Archaeospora VTX00005 | Soil | 0.522 | 0.001 |
| Archaeospora VTX00338 | Soil | 0.499 | 0.003 |
| Glomus VTX00309 | Soil | 0.477 | 0.001 |
| Glomus VTX00067 | Soil | 0.426 | 0.003 |

Supplementary Table 6. PERMANOVA of Bray-Curtis AMF community dissimilarities including the biochar packets (Sample) showing significant impact of Sample Type, Soil Type, and Treatment Type. Sample Type: the soil or biochar compartment. Soil Type: soils originating from Cranford, Vauxhall, Beaverlodge, or Olds. Treatment: Control; B: Biochar; B+M: Biochar and Manure; B+F: Biochar and Fertilizer. Df: degrees of freedom; SumsOfSqs: Sum of squares; F.Model: F value by permutation; R2: coefficient of determination; Pr(>F): p-values based on 1000 permutations (the lowest p-value being 0.001).

|  | Df | SumsOfSqs | MeanSqs | F.Model | R2 | Pr(>F) |
| --- | --- | --- | --- | --- | --- | --- |
| Sample | 1 | 3.6868 | 3.6868 | 17.7418 | 0.11854 | 0.001 |
| Soil | 3 | 5.0417 | 1.6806 | 8.0873 | 0.1621 | 0.001 |
| Treatment | 2 | 1.8795 | 0.9397 | 4.5222 | 0.06043 | 0.001 |
| Sample:Soil | 3 | 1.7306 | 0.5769 | 2.7761 | 0.05564 | 0.001 |
| Sample:Treatment | 2 | 1.442 | 0.721 | 3.4697 | 0.04636 | 0.001 |
| Soil:Treatment | 6 | 1.8625 | 0.3104 | 1.4938 | 0.05988 | 0.021 |
| Sample:Soil:Treatment | 6 | 1.7438 | 0.2906 | 1.3986 | 0.05607 | 0.038 |
| Residuals | 66 | 13.715 | 0.2078 |  | 0.44097 |  |
| Total | 89 | 31.1019 |  |  | 1 |  |

Supplementary Table 7. Pairwise PERMANOVA on Bray-Curtis distances across soil amendment treatments. Treatments include: Control; B: Biochar; B+M: Biochar and Manure; B+F: Biochar and Fertilizer. F.Model: F value by permutation; R2: coefficient of determination; p.value: p-values based on 1,000,000 permutations (the lowest p-value being 1.00E-06); p.adjusted: p-values as adjusted using a Bonferroni correction method.

| Comparisons | F.Model | R2 | p.value | p.adjusted |
| --- | --- | --- | --- | --- |
| Control: B | 0.513956 | 0.018025 | 8.19E-01 | 1 |
| Control: B+M | 2.29661 | 0.075804 | 3.29E-02 | 0.197604 |
| Control: B+F | 2.403816 | 0.09104 | 1.76E-02 | 0.105852 |
| B: B+M | 1.61544 | 0.051097 | 1.26E-01 | 0.757613 |
| B: B+F | 2.180377 | 0.077372 | 4.14E-02 | 0.248244 |
| B+M: B+F | 5.432156 | 0.172822 | 3.70E-05 | 0.000222 |
